# Biomarkers and Gene Signatures to Predict Durable Response to Pembrolizumab in Non-Small Cell Lung Cancer

**DOI:** 10.3390/cancers13153828

**Published:** 2021-07-29

**Authors:** Anello Marcello Poma, Rossella Bruno, Iacopo Pietrini, Greta Alì, Giulia Pasquini, Agnese Proietti, Enrico Vasile, Sabrina Cappelli, Antonio Chella, Gabriella Fontanini

**Affiliations:** 1 Department of Surgical, Medical, Molecular Pathology and Critical Area, University of Pisa, via Savi 10, 56126 Pisa, Italy; marcello.poma@med.unipi.it; 2 Unit of Pathological Anatomy, University Hospital of Pisa, via Roma 67, 56126 Pisa, Italy; rossella.bruno@for.unipi.it (R.B.); greta.ali@gmail.com (G.A.); agneseproietti@gmail.com (A.P.); 3 General Pathology, University of Pisa, via Savi 10, 56126 Pisa, Italy; iacopo.petrini@unipi.it; 4 Unit of Medical Oncology, San Jacopo Hospital of Pistoia, 51100 Pistoia, Italy; g.pasquini988@gmail.com; 5 Unit of Pneumology, University Hospital of Pisa, via Paradisa 2, 56126 Pisa, Italy; envasile@gmail.com (E.V.); sabrina.cap@tiscali.it (S.C.); anto.kell@tiscali.it (A.C.)

**Keywords:** lung cancer, immunotherapy, tumor microenvironment, predictive biomarkers, pembrolizumab, gene expression, PD-L1, XCL1, XCL2, CD8A, CD8B, EOMES

## Abstract

**Simple Summary:**

Not all patients with advanced or metastatic non-small cell lung cancer (NSCLC) respond to pembrolizumab, even if their tumor expresses PD-L1. This is a monocentric study aimed at identifying potential predictive biomarkers for pembrolizumab first-line treatment. Tumor microenvironment was characterized by gene expression analysis in 46 tumor samples from 25 NSCLC patients with and 21 without durable clinical benefit. As expected, patients achieving clinical benefit had a greater infiltration of immune cells. In particular, CD8 T-cell and NK cell scores were strongly associated with durable benefit. Single immune cell markers such as *XCL1/2* showed a high performance in predicting durable response to pembrolizumab with an AUC of 0.85. In the same series PD-L1 expression levels had an AUC equal to 0.61. Identified predictive biomarkers can improve patients’ selection, thus optimizing treatment definition.

**Abstract:**

Pembrolizumab has been approved as first-line treatment for advanced Non-small cell lung cancer (NSCLC) patients with tumors expressing PD-L1 and in the absence of other targetable alterations. However, not all patients that meet these criteria have a durable benefit. In this monocentric study, we aimed at refining the selection of patients based on the expression of immune genes. Forty-six consecutive advanced NSCLC patients treated with pembrolizumab in first-line setting were enrolled. The expression levels of 770 genes involved in the regulation of the immune system was analysed by the nanoString system. PD-L1 expression was evaluated by immunohistochemistry. Patients with durable clinical benefit had a greater infiltration of cytotoxic cells, exhausted CD8, B-cells, CD45, T-cells, CD8 T-cells and NK cells. Immune cell scores such as CD8 T-cell and NK cell were good predictors of durable response with an AUC of 0.82. Among the immune cell markers, *XCL1/2* showed the better performance in predicting durable benefit to pembrolizumab, with an AUC of 0.85. Additionally, *CD8A*, *CD8B* and *EOMES* showed a high specificity (>0.86) in identifying patients with a good response to treatment. In the same series, PD-L1 expression levels had an AUC of 0.61. The characterization of tumor microenvironment, even with the use of single markers, can improve patients’ selection for pembrolizumab treatment.

## 1. Introduction

Immunotherapy improved the treatment options for advanced and metastatic non-small cell lung cancer (NSCLC) without actionable driver mutations [1,2]. In particular, the use of checkpoint inhibitors (ICIs) against anti-programed death 1 (PD-1) (pembrolizumab and nivolumab) and anti-programed death ligand 1 (PD-L1) (atezolizumab) has considerably improved patients’ outcome [2,3,4,5,6,7]. Among them, pembrolizumab was the first ICI approved as single agent for the first-line treatment of NSCLC expressing PD-L1 [3,4,8]. Although pembrolizumab proved to be more effective than conventional chemotherapy, the overall response rate is not completely satisfying [3,8]. Nowadays, the evaluation of PD-L1 expression by immunohistochemistry (IHC) is the current standard to drive the selection of patient for pembrolizumab administration [1]. Although a high PD-L1 expression has been associated with better responses to pembrolizumab, the sensitivity and specificity of this biomarker is limited. In the Keynote 024 trial, the overall response rate was 45% even if all the enrolled patients had PD-L1 levels greater than 50% [4]. In the Keynote 042 trial, 118 out of 299 patients (39%) with a PD-L1 tumor proportion score (TPS) greater than or equal to 50% had an objective response to treatment [8]. Considering the complexity and the heterogeneity of tumor-immune system interaction, it is unlikely that PD-L1 alone can discriminate immunogenic from non-immunogenic tumors. In fact, tumor cells can use also alternative checkpoints (e.g., CTLA4, LAG3, IDO, VISTA) and escape mechanisms [9,10,11].

Other predictive biomarkers have been proposed, mainly divided into two groups: biomarkers associated with high tumor antigenicity, including tumor mutational burden (TMB) and microsatellite instability (MSI-H)/mismatch repair deficiency (MMRd); and biomarkers associated with intense tumor inflammation, including tumor infiltrating lymphocytes and immune gene signatures [2,10].

A high TMB has been correlated with a better response to ICI in lung cancer, and it has been reported as independent from PD-L1 expression levels [5,12]. Recently, the Food and Drug Administration (FDA) approved pembrolizumab for adults and pediatric patients with TMB greater than 10 mutations/megabase that have progressed following prior treatments. However, the lack of technical standardization (whole exome or targeted panels) and of a universally accepted cut-off, together with the high costs and execution time has always limited its introduction in clinical practice [13]. In the same way, MSI-H and MMRd can predict a high neoantigen load; they have been approved by the FDA as agnostic biomarkers to treat with pembrolizumab patients with inoperable or metastatic solid tumor that have progressed following prior treatments [14]. However, the prevalence of MSI-H in NSCLC is very low (about 0.6%), and data for ICIs in NSCLC with MSI-H are still poor [15].

Tumor infiltrating lymphocytes (TIL) are directly correlated with improved survival in NSCLC, and the presence of cluster of differentiation 8 (CD8) and cytotoxic T lymphocytes in the tumor stroma has been associated with a good ICI response [16,17,18]. Besides the assessment of TIL, inflamed and non-inflamed tumors can be discriminated also by the analysis of immune gene expression signatures. Among suggested gene signatures, Ayers and collaborators defined an 18-mRNA gene expression panel (i.e., the T cell-inflamed gene expression profile (GEP) signature) related to IFN-γ and activated T-cells associated with response to pembrolizumab across different tumor types [19]. More recently, Hwang et al. by evaluating the expression of about 395 immune-related genes identified two signatures “M1” and “peripheral T cell” and two biomarkers (CD137 and PSMB9) able to discriminate NSCLC patients achieving durable clinical benefit to pembrolizumab treatment [20]. Likewise, two studies reported immune cell scores as predictors of durable benefit from checkpoint inhibitors using the nCounter technology [21,22].

Although the promising results related to both characterization of tumor antigenicity and inflammation status, no biomarkers other than PD-L1 have been introduced in NSCLC to select patients for first-line treatment with pembrolizumab [1]. In some cases, prospective validation and a clear correlation with the progression free survival (PFS) and overall survival (OS) are still missing. Moreover, the translation of some tests (i.e., TMB or gene signatures) in routine procedures is challenging. In fact, most of the NSCLC patients in advanced stage of disease are no candidates for surgery, and small biopsies or cytology samples are the only available diagnostic material in more than 50% of cases [23,24]. In addition, precision medicine in NSCLC includes both ICI and targeted therapies, requiring the characterization of several predictive biomarkers [25,26]. As a consequence, the biological material is almost always a limiting factor for molecular testing in advanced or metastatic NSCLC.

In this context, the search for predictive biomarkers for ICI is still ongoing and strictly necessary.

In this study, we investigated the tumor microenvironment of a consecutive series of NSCLC patients treated with first-line pembrolizumab in the absence of other targetable alterations. The expression profile of 770 genes involved in the regulation of the immune system was evaluated in order to identify predictive biomarkers applicable in clinical practice able to improve patients’ selection.

## 2. Materials and Methods

### 2.1. Study Population

In this study, 46 consecutive patients with advanced or metastatic NSCLC (including both adenocarcinoma and squamous cell carcinoma) were enrolled at the University Hospital of Pisa. All patients were negative for targetable alterations within *EGFR*, *ALK*, *ROS1*, *BRAF*, *MET* and *ERBB2* genes, and all tumors expressed PD-L1 in more than 50% of tumor cells. Enrolled patients received pembrolizumab as single agent first-line treatment. 

Patients’ clinical evaluation has been performed every 3 months from the beginning of treatment and responses were defined according to the Response Evaluation Criteria in the Solid Tumors (RECIST) guidelines, version 1.1. Treatment was continued until disease progression or intolerable toxicity, physician’s or patient’s decision. Patients were divided into two groups: those achieving durable clinical benefit (DCB) defined as progression-free interval >6 months, and non-durable clinical benefit (NCB).

For all patients, formalin-fixed paraffin-embedded (FFPE) tumor biopsies or cell-blocks from fine needle aspiration, brushing and pleural effusion specimens obtained before pembrolizumab treatment were used for gene expression analysis. 

This study was conducted according to the guidelines of the Declaration of Helsinki and it was approved by the local Ethics Committee. 

### 2.2. PD-L1 and Gene Expression Tests

The expression of PD-L1 was determined by IHC. In details, 3µ-thick FFPE sections were incubated with the rabbit monoclonal primary antibody SP263 (Roche, Monza, Italy). Staining was performed by the Ventana Benchmark Ultra staining platform (Ventana Medical Systems, Tucson, AZ, USA). The percentage of PD-L1 expression on tumor cells was blindly determined by two expert pathologists and the median value was considered. The PD-L1 expression was evaluated by TPS, which is defined as the percentage of viable tumor cells with partial or complete membrane staining of any intensity (≥1+) relative to all viable tumor cells in the examined section [27]. At least 100 viable tumor cells must be present for the evaluation.

For all samples, tumor cell percentage was estimated independently by two expert pathologists and tumor component was enriched by manual macrodissection before nucleic acid extraction. In detail, total RNA was purified from three-to-four unstained FFPE sections (5 µm-thick) using the Qiagen RNeasy FFPE kit (Qiagen, Hilden, Germany), and according to the manufacturer’s procedures. RNA quality and concentration were assessed using an Xpose spectrophotometer (Trinean, Gentbrugge, Belgium). About 100 ng of total RNA were used for gene expression analysis using the nCounter system (nanoString Technologies, Seattle, WA, USA). Total RNA was hybridized with capture and reporter probes at 60 °C for 18 h; cleanup of samples and counts of digital reports were performed as described by the manufacturer (nanoString Technologies).

Expression levels of genes included in the nanoString PanCancer IO 360 Panel code set were evaluated. This code set is a 770-gene expression panel, allowing the evaluation of different immune pathways [28].

### 2.3. Data Analyses and Statistics

Raw counts were normalized using the Advanced Analysis module of the nSolver v.4.0 (nanoString Technologies). Normalized expression counts were log2-transformed, and genes with raw counts as low as 20 were omitted from further analyses. Differentially expressed genes (DEG) were computed by a mixture negative binomial model, a simplified negative binomial model or a log-linear model according to the best converging algorithm for each gene. Patients with NCB were used as baseline. *p*-values were adjusted with the Benjamini–Yekutieli method, and a false discovery rate (FDR) below 0.05 was deemed significant. Cell type scores were computed using the method described by Danaher [29]; only cell types with at least two specific markers were considered. The lists of transcripts used to compute cell type scores are reported in Supplementary Table S1. DEG and cell type scores were computed using the Advanced Analysis module of the nSolver. Differences among cell types were computed by the Welch’s *t*-test. A *p*-value of 0.05 was used as significance cut-off. The Gene Set Enrichment Analysis (GSEA) was run using the fold-change-ranked gene list and a minimum of ten genes per gene set as cut-off; the analysis was performed following the procedures of clusterProfiler Bioconductor package v.3.13 and using the Hallmark collection as reference. The Benjamini–Hochberg procedure was used to adjust *p*-values, and an FDR below 0.05 was considered significant. Correlation among continuous variables was tested by Pearson’s method using a *p*-value of 0.05 as significance cut-off. The T cell-inflamed gene expression profile (GEP) [19] was calculated by averaging the expression of 16 out of 18 genes of the original signature that were analyzed by the nanoString assay, namely *IL2RG*, *CXCR6*, *CD3D*, *CD2*, *HLA-DRA*, *CCL5*, *NKG7*, *CD3E*, *HLA-E*, *GZMB*, *GZMK*, *CXCL13*, *CXCL10*, *IDO1*, *LAG3* and *STAT1*. To identify predictors of DCB, the receiver operating characteristics (ROC) curves analysis was performed following the procedures of pROC R package v. 1.17.0.1. The 95% confidence intervals (CI) were calculated by 2000 bootstrap replicates, and the best cut-off was computed using the Youden’s J statistics. Univariate survival analysis was performed by Cox regression using log2 gene expression levels as continuous variables and following the procedures of the survival R package v.3.2-11. All analyses and plots were generated in R environment (https://www.r-project.org/, v.4.1.0, last accessed on 18 June 2021) unless otherwise specified.

## 3. Results

### 3.1. Population

Forty-six patients fulfilled all the inclusion criteria and were enrolled in this study. Characteristics of study population are summarized in Table 1.

### 3.2. Immune Activation in Patients with DCB

**Figure 1:**
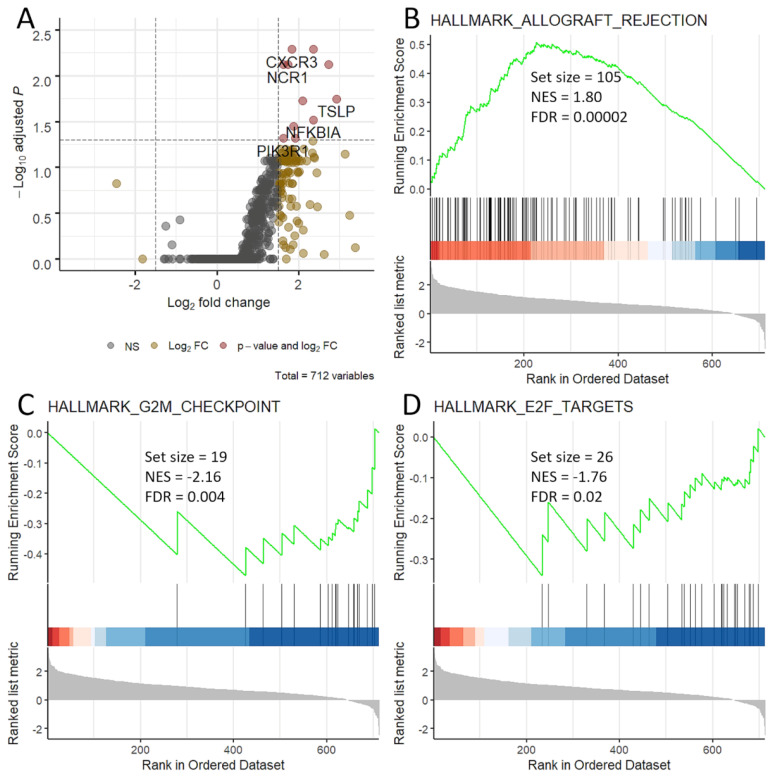
Gene expression differences between patients with and without durable clinical benefit to pembrolizumab. Patients with non-durable clinical benefit were used as baseline. (**A**) Volcano plot. Log2 fold changes (*x*-axis) and −Log10 of false discovery rate (*y*-axis) were plotted. Red denotes genes that are significantly different. (**B**–**D**) Gene sets differentially activated based on GSEA analysis; in detail, Allograft rejection (**B**) is activated in patients with durable benefit, while G2M checkpoint (**C**) and E2F targets (**D**) are activated in patients who progressed early. NES, normalized enrichment score; FDR, false discovery rate.

After normalization, 712 genes were considered. Contrasting patients with DCB vs. NCB, there were marked gene expression imbalances; in fact, 330 genes (46% of total) had a *p* < 0.05 with a great predominance of up-regulation. However, after adjustment for multiple comparisons, only 11 genes were significantly up-regulated (FDR < 0.05), namely *CXCR3*, *BCL2*, *NCR1*, *CXCL13*, *FASLG*, *TSLP*, *XCL1/2*, *NFKBIA*, *CCL5*, *PIK3R1* and *IL11RA* (Figure 1A). The complete results of DEG analysis is reported in Supplementary Table S2. Patients with DCB had a significant activation of the Allograft Rejection gene set, while the G2M Checkpoint and E2F Targets gene sets were activated in patients with NCB (Figure 1B–D).

**Figure 2:**
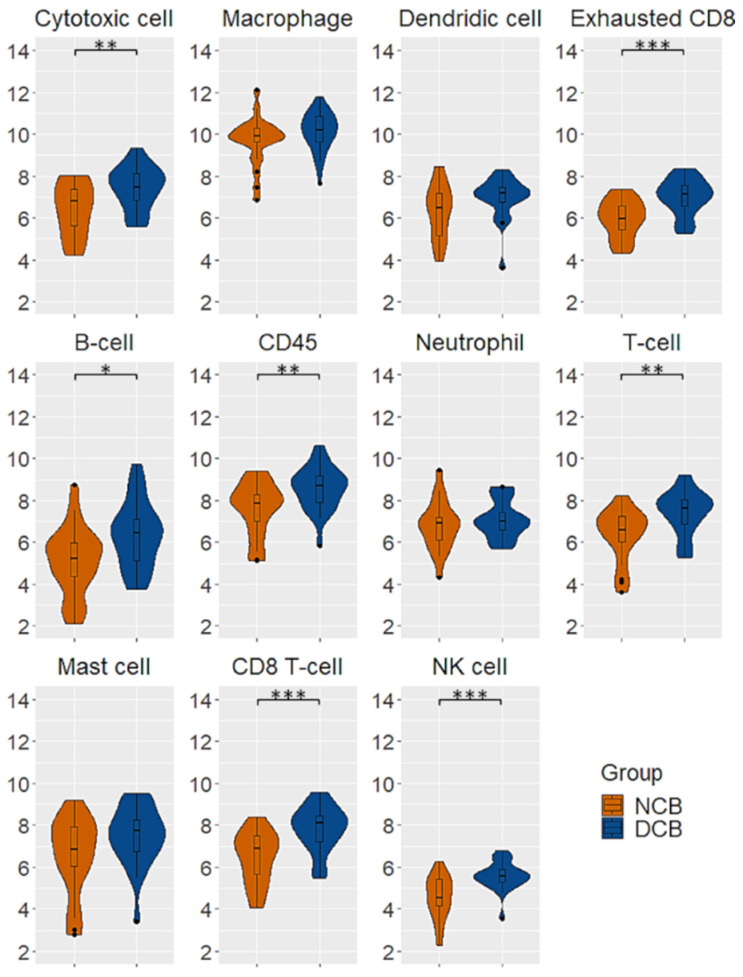
Immune cell scores differences between patients with and without durable clinical benefit to pembrolizumab. Patients with durable benefit had higher levels of cytotoxic cell, exhausted CD8, B-cell, CD45, T-cell, CD8 T-cell and NK cell infiltration. * *p* < 0.05; ** *p* < 0.01; *** *p* < 0.001.

As regards immune cells, patients with DCB had a greater infiltration of cytotoxic cells, exhausted CD8, B-cells, CD45, T-cells, CD8 T-cells and NK cells (Figure 2).

### 3.3. Correlation of PD-L1 and RAS/RAF Mutation with Immune Cells and Durable Response

PD-L1 IHC levels positively correlated with *CD274* transcript abundance (R = 0.64, *p* = 0.0001). PD-L1 levels were significantly higher in tumors with mutation in *BRAF* (non-V600E) or the *RAS* genes (*p* = 0.04). PD-L1 levels were negatively correlated with mast cell score (*p* = 0.006, R = −0.40), but were not significantly correlated with others cell types. Similarly, the presence of *RAS/RAF* mutations was not associated with immune cells abundance. In addition, neither *RAS/RAF* mutations nor the exact percentage of PD-L1 accurately predicted a durable benefit to pembrolizumab (area under the curve, AUC 0.54, 95% CI: 0.40–0.68, and AUC 0.61, 95% CI: 0.44–0.78, respectively).

### 3.4. Cell Type Abundance and Markers to Predict Durable Benefit to Pembrolizumab

Almost all cell type scores had a better performance in predicting durable benefit to pembrolizumab compared to the exact PD-L1 percentage. In particular, exhausted CD8, CD8 T-cell and NK cell abundance had an AUC > 0.80 (Table 2). Similarly, Ayers’ T cell-inflamed GEP signature had an AUC of 0.80 (95% CI 0.67–0.91) and was associated with a better PFS (*p* = 0.0001, HR = 0.51, 95% CI 0.36–0.74).

**Figure 3:**
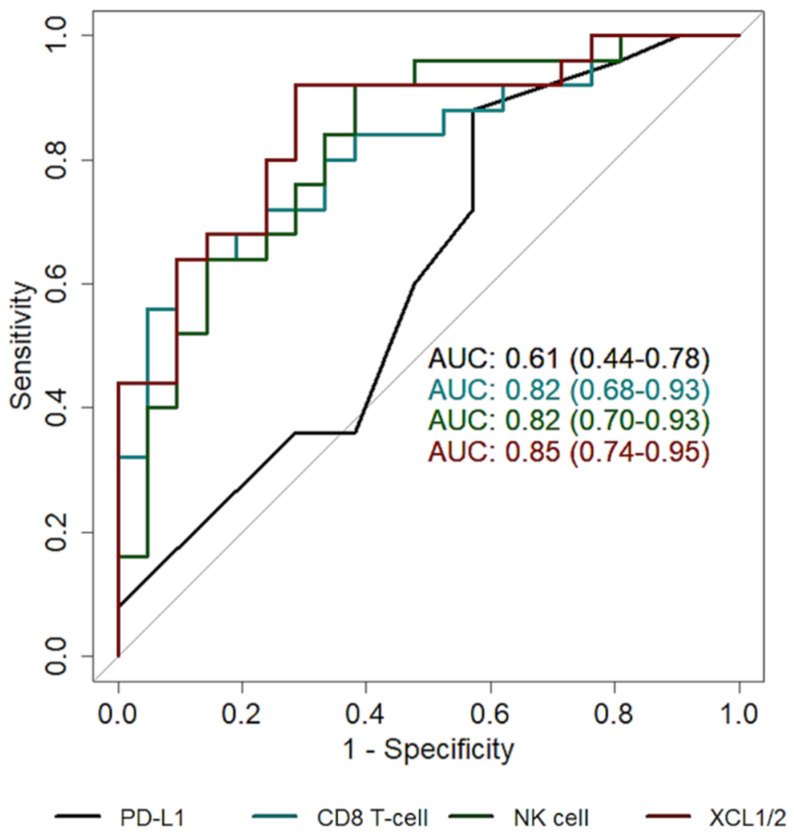
Prediction of durable response. ROC curves of PD-L1 (black), CD8 T-cell (cyan), NK cell (green) and *XCL1/2* (red).

In order to find useful markers associated with durable benefit, we considered the marker genes of the cell types with AUC greater than 0.80, namely *CD244*, *LAG3*, *EOMES* and *PTGER4* for exhausted CD8; *CD8A* and *CD8B* for CD8 T-cell; *XCL1/2* and *NCR1* for NK cell. Among these, *XCL1/2* showed the better performance in predicting durable benefit to pembrolizumab, with an AUC of 0.85 (95% CI: 0.74–0.95, Table 3, Figure 3). 

## 4. Discussion

After the superiority to standard chemotherapy showed in the KEYNOTE-024 trial, pembrolizumab was the first anti-PD-1 drug approved as single agent for first-line setting in advanced NSCLC [4]. The eligibility for pembrolizumab treatment requires the absence of targetable alterations and the expression of PD-L1 evaluated by IHC. At the first approval, the PD-L1 cut-off was set as 50% of positive tumor cells. Since a significant benefit was observed also in a proportion of patients with tumor expressing lower levels of PD-L1, the cut-off was than lowered to 1% [30]. Despite the PD-L1-driven selection of patients, the overall response rates are not completely satisfying, and only a small proportion of patients achieve a durable benefit [4,8]. In this study we analysed the tumor microenvironment in 46 consecutive advanced NSCLC patients who received first-line pembrolizumab, and whose tumors express more than 50% of PD-L1. We divided patients into two groups: patients who achieved a durable clinical benefit (DCB, i.e., more than 6 months) and those who progressed within 6 months (non-durable clinical benefit, NCB). Overall, 25 patients (54.3%) had a DCB with a median PFS of 30.6 months (95% CI 22.4 to not reached), while 21 patients (45.7%) were included in the NCB group with a median PFS of 2.8 (95% CI 2.0 to 4.2). Indeed, a median PFS of 30 months is exceptionally high even referred to a DCB group. The selection based on the 50% PD-L1 cut-off as well as the presence of stage III patients (about one third) might have influenced these data. Patients in the DCB group had a substantial up-regulation of immune genes. On the other hand, the activation of the G2M checkpoint and E2F targets signatures was enriched in patients who progressed early. Although higher G2M and E2F targets scores had been associated with a greater PD-L1 expression in NSCLC [31], the G2M checkpoint activation in pancreatic and breast cancer was linked to Th2 response [32,33], which is typically associated with the promotion of tumor growth and the repression of anti-tumor immunity. Similarly, CDK4/6 inhibition, which suppress the activity of E2F targets, demonstrated to trigger anti-tumor immunity, thus uncovering a new function of E2F targets beyond the regulation of cell cycle [34,35]. *KRAS* and *BRAF* non-V600E mutations are more frequent in smokers and have been reported as correlated with an immunogenic tumor microenvironment [36]. However, in our series, neither the PD-L1 TPS nor the presence of *RAS/RAF* mutations were associated with a DCB. In addition, both PD-L1 and *RAS/RAF* mutations did not correlate with the infiltration of immune cells, thus suggesting that immune cell scores are independent biomarkers. 

Notably, almost all immune cell scores, especially CD8 T-cell and NK cells, showed a good performance in predicting a DCB. The results are slightly different from those obtained by Budczies and colleagues, who have reported that only B-cell and total TIL abundance predict benefit from immune checkpoint blockade [21]. These differences could be due to randomness, which is relevant in presence of small sample size, but the 50% cut-off selection of the present study might account for higher levels of immune infiltrates. On the other hand, Frigola and colleagues have found several immune scores associated with durable benefit to checkpoint inhibitors, including B-cell, NK, CD8 and Treg [22]. Of note, Ayers T-cell-inflamed GEP signature was strongly associated with DCB, similarly to CD8 T-cell and NK cell. CD8 T-cell and NK cell infiltration have already been associated with improved survival of lung cancer patients and with a longer PFS after treatment with ICIs [22,37]. These evidences suggest that the use of gene signatures for the characterization of the immune infiltrate could be useful to refine the selection of patients for ICIs administration. Indeed, the analysis of gene signatures should be harmonized since the type of the platform, the markers used and the algorithm can influence the outcome. For this reason, single immune cell markers combined with PD-L1 could be a simple and effective strategy. In our series, *XCL1/2* showed the highest performance in predicting a DCB. *XCL1* and *XCL2* are chemokines produced by NK cells and are essential for the recruitment of conventional type I dendritic cells (cDC1) [38]. Along with the cross-priming of CD8 T-cell, cDC1 exert several pro-inflammatory functions within the tumor microenvironment, which result in an enhanced anti-tumor immunity thus increasing the responsiveness to ICIs [39,40]. Other markers, such as *CD8A* and *CD8B*, which are specific for CD8 T-cell, and *EOMES*—a marker of exhausted CD8—showed a very high specificity in predicting a DCB. CD8A was already associated with a prolonged survival and a longer PFS after treatment with the anti-PD-1 nivolumab [41]. In addition, EOMES was suggested—alongside with CD8/CD4, CD69 and CD45RO—as a marker of memory T-cells that associate with an improved response to anti-PD-1 blockade, both as single agent or combination therapy [42]. The other immune markers, namely *CD244*, *LAG3*, *PTGER4* and *NCR1* showed a lower performance in predicting durable benefit. Nevertheless, these genes are expressed mostly by immune cells, and they play important roles in modulating the immune response. For instance, *CD244* encodes for a receptor expressed in cytotoxic cells that is essential for tuning the effector activity [43]. *LAG3* is expressed on exhausted T-cells and represents an alternative immune checkpoint to PD1/PD-L1 axis. Targeting *LAG3* is therefore an attractive strategy to overcome immune resistance [44]. *PTGER4* encodes for a receptor of prostaglandin E2, which is not exclusively expressed by immune cells. However, this receptor is crucial for the regulation of immune response as suggested by its involvement in chronic immune diseases [45,46]. Finally, *NCR1* is a marker of NK cell activation, but, under chronic viral infection, it was reported that NCR1-FcRγ complex dampens T-cell activity thus favoring chronic infection [47,48].

There are some limitations that should be acknowledged. First, the sample size of the study is limited; moreover, all cases had more than 50% of PD-L1 since they were gathered before the cut-off change for pembrolizumab administration. Nevertheless, our findings are based on a consecutive real-life series and warrant further validation also in the light of the 1% PD-L1 cut-off.

In closing, we reported that just over 50% of advanced NSCLC achieved a durable clinical benefit after first-line treatment with pembrolizumab despite the selection based on the 50% PD-L1 TPS. The analysis of the tumor immune infiltrate can assist the selection, and even the use of single markers of CD8 T-cell and NK cells can identify patients that achieve a durable benefit.

## 5. Conclusions

The PD-L1–based selection of patients for pembrolizumab administration as single agent in first-line setting is not satisfactory. The analysis of immune cell infiltrate can refine the identification of patients likely to achieve a durable clinical benefit. The use of single CD8 T-cell and NK cell markers such as *CD8A/B* and *XCL1/2* could be a simple and effective strategy for clinical practice.

## Figures and Tables

**Table 1:** Clinical features of patients according to response group to pembrolizumab.

Clinical Features	DCB, *n* (%) (Total 25, 54.3%)	NCB, *n* (%) (Total 21, 45.7%)	*p*-Value
Age (years)			0.29
mean ± SD	69.2 ± 7.7	66.6 ± 8.5	
Gender			1
Male	19 (76.0)	16 (76.2)	
Histology			0.79
ADC	14 (56.0)	11 (52.4)	
SCC	7 (28.0)	5 (23.8)	
NOS	4 (16.0)	5 (23.8)	
Stage			0.57
III	9 (36.0)	5 (23.8)	
IV	16 (64.0)	16 (76.2)	
Smoking status			0.73
smoker	6 (24.0)	3 (14.3)	
former	18 (72.0)	17 (80.9)	
never	1 (4.0)	1 (4.8)	
ECOG PS			0.12
0	6 (24.0)	3 (14.3)	
1	17 (68.0)	11 (52.4)	
2	2 (8.0)	7 (33.3)	
Toxicity			0.04
yes	14 (56.0)	5 (23.8)	
no	11 (44.0)	16 (76.2)	
*RAS* or *BRAF(non-V600E)* mutation			0.83
yes	10 (40.0)	10 (47.6)	
no	15 (60.0)	11 (52.4)	
PFS (months)			<0.0001
median (CI)	30.6 (22.4–NR)	2.8 (2.0–4.2)	
OS (months)			<0.0001
median (CI)	NR (27.5–NR)	5.6 (3.6–13.5)	

DCB, durable clinical benefit; NCB, non-durable clinical benefit; SD, standard deviation; ADC, adenocarcinoma; SCC, squamous cell carcinoma; NOS, not otherwise specified; ECOG PS, Eastern Cooperative Oncology Group performance status; PFS, progression-free survival; OS, overall survival; NR, not reached.

**Table 2:** Usefulness of cell type scores to predict durable response to pembrolizumab.

Cell Type	AUC (95% CI)	*p*-Value *	HR (95% CI)	*p*-Value *
Cytotoxic cell	0.74 (0.59–0.87)	0.003	0.59 (0.42–0.82)	0.002
Macrophage	0.63 (0.46–0.79)	0.18	0.78 (0.54–1.27)	0.2
Dendritic cell	0.67 (0.51–0.83)	0.06	0.69 (0.50–0.94)	0.02
Exhausted CD8	0.80 (0.67–0.92)	0.0002	0.46 (0.30–0.70)	0.0004
B-cell	0.71 (0.56–0.86)	0.01	0.72 (0.54–0.94)	0.02
CD45	0.71 (0.55–0.85)	0.008	0.65 (0.47–0.89)	0.007
Neutrophil	0.58 (0.42–0.74)	0.38	0.90 (0.61–1.34)	0.6
T-cell	0.76 (0.62–0.89)	0.004	0.60 (0.44–0.83)	0.002
Mast cell	0.63 (0.46–0.79)	0.07	0.77 (0.61–0.96)	0.02
CD8 T-cell	0.82 (0.68–0.93)	0.0003	0.56 (0.41–0.76)	0.0003
NK cell	0.82 (0.70–0.93)	0.0003	0.48 (0.33–0.71)	0.0003
PD-L1 **	0.61 (0.44–0.78)	0.20	0.99 (0.96–1.01)	0.32

* *p*-value refers to difference between DCB and NCB. ** tumor proportion score; AUC, area under the curve; CI, confi-dence interval; HR, hazard ratio.

**Table 3:** Usefulness of cell type markers to predict durable response to pembrolizumab.

Marker	Cell Type	AUC (95% CI)	Sensitivity ^§^ (95% CI) *	Specificity ^§^ (95% CI) *	Accuracy (95% CI) *	Correlation with PD-L1 **, R *** (*p*-Value)	HR (95% CI), *p*-Value
*CD244*	exhausted CD8	0.68 (0.52–0.84)	0.84 (0.44–1)	0.57 (0.29–0.95)	0.72 (0.61–0.83)	0.06 (0.71)	0.65 (0.48–0.89), 0.007
*LAG3*	exhausted CD8	0.75 (0.61–0.89)	0.68 (0.44–0.92)	0.86 (0.57–1)	0.76 (0.65–0.87)	0.24 (0.11)	0.66 (0.48–0.90), 0.009
*EOMES*	exhausted CD8	0.82 (0.69–0.92)	0.68 (0.44–1)	0.95 (0.48–1)	0.78 (0.67–0.89)	0.25 (0.09)	0.48 (0.34–0.67), 0.00002
*PTGER4*	exhausted CD8	0.75 (0.61–0.87)	0.64 (0.40–1)	0.90 (0.43–1)	0.74 (0.63–0.85)	0.03 (0.82)	0.47 (0.27–0.81), 0.007
*CD8A*	CD8 T-cell	0.79 (0.66–0.91)	0.68 (0.48–0.92)	0.95 (0.71–1)	0.78 (0.70–0.89)	0.10 (0.51)	0.61 (0.46–0.82), 0.001
*CD8B*	CD8 T-cell	0.82 (0.69–0.93)	0.76 (0.56–0.96)	0.86 (0.62–1)	0.80 (0.70–0.91)	0.13 (0.38)	0.52 (0.38–0.73), 0.0001
*XCL1/2*	NK cell	0.85 (0.74–0.95)	0.88 (0.56–1)	0.81 (0.57–1)	0.83 (0.74–0.93)	−0.08 (0.62)	0.48 (0.34–0.66), 0.000007
*NCR1*	NK cell	0.67 (0.51–0.82)	0.88 (0.28–1)	0.57 (0.38–1)	0.72 (0.59–0.85)	0.17 (0.26)	0.75 (0.53–1.07), 0.12
PD-L1 **	NA	0.61 (0.44–0.78)	0.88 (0.20–1)	0.43 (0.19–1)	0.67 (0.54–0.78)	NA	0.99 (0.96–1.01), 0.32

* Sensitivity, specificity and accuracy were computed using the better cut-off according to the Youden’s J statistics. ** Exact percentage. *** R, Pearson’s correlation. ^§^ Sensitivity and specificity refer to the best cut-off according to the Youden J statistics. AUC, area under the curve; CI, confidence interval; NA, not applicable; HR, hazard ratio.

## Data Availability

The data presented in this study are available on request from the corresponding author.

## Supplementary Materials

The following are available online at https://www.mdpi.com/article/10.3390/cancers13153828/s1, Table S1: Lists of transcripts used to compute cell type scores; Table S2: Differentially expressed genes. In Table S2 are reported differentially expressed genes between patients achieving durable clinical benefit and non-clinical benefit after first line pembrolizumab treatment.
